# Measuring the Impact of the COVID-19 Pandemic on Health Behaviors and Health Care Utilization in Rural and Urban Patients with Cancer and Cancer Survivors

**DOI:** 10.1158/2767-9764.CRC-22-0386

**Published:** 2023-02-07

**Authors:** Allison Cole, C. Holly A. Andrilla, Davis Patterson, Sarah Davidson, Jason Mendoza

**Affiliations:** 1 Department of Family Medicine, University of Washington, Seattle, Washington.; 2 University of Washington, Seattle, Washington.; 3 Fred Hutchinson Cancer Center, Seattle, Washington.

## Abstract

Health care access and health behaviors differ between those living in urban and rural communities and contribute to inequitable cancer health outcomes. The COVID-19 pandemic led to significant disruptions in daily life and health care delivery. This cross-sectional survey aimed to measure the impact of the COVID-19 pandemic on the health behaviors of patients with cancer and survivors, comparing outcomes for urban and rural respondents. Survey was administered from January 2021 to June 2021 to patients with cancer or survivors (treated within the last 5 years) at one of six cancer centers in Washington and Idaho. Respondent ZIP code was used to assess rurality using rural-urban commuting area designation. A total of 515 rural (43.5% of those contacted) and 146 urban (40% of those contacted) patients with cancer and survivors participated. Few differences between urban and rural patients with cancer and cancer survivors were noted. Rural residents were older (69.2 vs. 66.9 years). Rural respondents had higher mean alcohol consumption than urban respondents (4.4 vs. 2.7 drinks per week). A total of 12.2% of those who reported drinking in the last 30 days also reported increased alcohol consumption since the start of the pandemic, with no difference in reported increased alcohol consumption in rural versus urban respondents. A total of 38.5% reported decreased physical activity. A total of 20.5% reported canceling or delaying cancer care due to the COVID-19 pandemic. Delays in cancer health care services and worsening health behaviors due to the COVID-19 pandemic may contribute to poorer health outcomes, with few differences between rural and urban patients with cancer and cancer survivors.

**Significance:**

COVID19 led to significant disruptions in health care access and daily life. Rural communities experience barriers to healthy behaviors and health care access that contribute to poorer cancer outcomes, compared with urban populations. The impact of COVID19 on rural and urban patients with cancer and cancer survivors has not been assessed.

## Introduction

The COVID-19 pandemic and subsequent public health mitigation strategies have led to significant disruptions in daily life as well as changes to health care access and health care delivery. In April 2020, the Centers for Medicare & Medicaid Services recommended U.S. health care systems defer nonessential surgeries, procedures, and medical services to conserve critical health care resources and limit exposure of patients and staff to COVID-19 (1). By June 2020, among a representative sample of U.S. adults, 41% reported having delayed or avoided medical care due concerns related to COVID-19 (2). And in September 2020, almost half of U.S. adults reported delaying health care, primarily due to fear of COVID-19 infection (3). COVID-19 also led to significant changes in health behaviors, such as reduction in physical activity (reported by 24% of adults internationally) (4) and increased alcohol consumption (5, 6).

Cancer care in particular experienced setbacks as screening programs and diagnostic services were decreased or suspended (7). COVID-19 has also resulted in the suspension of many clinical trials—the cessation of which could have long-lasting financial and health consequences for patients with cancer (7).

Even though 19% of the U.S. population resides in rural communities, barriers to health care access have contributed to longstanding and persistent cancer health disparities in rural populations (8). From 2009 to 2013, compared with urban residents, the incidence of cancer diagnosis (460/100,000 rural vs. 447/100,000 urban) and the cancer mortality rate (182/100,000 rural vs. 166/100,000 urban) among rural residents was higher (9). Residents of rural counties are more likely to be diagnosed with late-stage lung, breast, cervix, and colorectal cancers compared with residents of urban counties (10). Cancer treatment outcomes for rural residents are worse than for urban residents: rural patients diagnosed with stage I non–small cell lung cancer underwent fewer surgeries (69% vs. 75%) and had significantly reduced median survival (40 vs. 52 months) compared with the most urban patients. Rural patients with breast cancer are less likely than urban patients with breast cancer to receive radiation and surgery with radiation (11). Compared with urban populations, rural residents are less likely to receive recommended radiotherapy for cancer (12), appropriate prostate cancer treatment (13) and appropriate colorectal cancer treatment (14). Rural-residing cancer survivors report worse overall health, more psychologic distress, and higher health-related unemployment compared with urban cancer survivors (15). Rural colorectal cancer survivors are twice as likely as urban survivors to report treatment-related financial hardship (16). In light of these pre-COVID-19 rural/urban disparities, there is urgent need to better understand the impact of social distancing strategies and health care reorganization efforts on both rural and urban patients with cancer and cancer survivors (17).

The objective of this study is to assess the degree to which patients with cancer and survivors in both urban and rural communities reported negative changes in health behaviors or delays in cancer-related health care. The findings of this study are useful in understanding the potential health consequences of the COVID-19 pandemic on patients with cancer and cancer survivors and can be used to guide development or tailoring of interventions to improve health behaviors or health care access in these vulnerable populations.

## Materials and Methods

### Study Design, Setting, and Participants

We conducted a cross-sectional survey, administered via email, telephone, or mail to adult patients with cancer and survivors from five cancer center sites located in Washington and Idaho (Sequim, WA; Seattle, WA; Marysville, WA; Wenatchee, WA; Lewiston, ID) selected from the Seattle Cancer Care Alliance's Network program.

Each center provided the University of Washington (UW) research team the names and contact information (mailing address, telephone number, and email address if available) of potentially eligible participants: individuals ages 18 or older living in Washington or Idaho, who were either receiving or had received cancer treatment in the previous 5 years.

We used rural-urban commuting area (RUCA) codes to classify residence ZIP codes into two geographic categories: urban and rural. RUCA codes with a primary digit of 1, 2, or 3 as well as codes 4.1, 5.1, 7.1, 8.1, and 10.1 were classified as urban. All other RUCA codes were classified rural. The primary digit of the RUCA codes categorizes metropolitan and nonmetropolitan areas based on the size and direction of the primary commuting flow. Secondary commuting flows and connections among rural and urban places are captured in the second digit of the RUCA code (18). To maximize rural participation, we attempted to contact and enroll all potential rural participants (*n* = 1,210). We randomly selected a subset of 337 urban patients with cancer and cancer survivors for survey invitation. Survey participants provided informed consent prior to participation and all research procedures were consistent with the U.S. Common Rule. The UW Human Subjects Division reviewed this study and determined it to be exempt human subject research.

### Data Collection

We administered the survey from January to June 2021 using Research Electronic Data Capture (REDCap). For individuals with an email address, an email study invitation described the survey as well as potential risks and benefits and provided a participant-specific internet survey link. We emailed up to eight reminders over approximately 1 month to complete the questionnaire electronically, followed by up to four telephone contacts and mailing of a hard copy version to nonrespondents. We attempted to contact those with no available email address via phone either to obtain an email address, administer the questionnaire by telephone, or confirm a mailing address to mail the questionnaire. For potential participants who identified as Spanish speaking, a bilingual research coordinator administered the survey over the telephone in Spanish. Participants were offered a $15 electronic gift card for participation.

### Measures

We used survey questions and measures adopted from a larger study conducted in collaboration with nine NCI funded sites (Table 1) to assess psychosocial and behavioral impacts of COVID-19 in our patients with cancer and survivors. Core measures focused on work and employment, housing/home life, social activities, emotional well being, physical health, and health behaviors.

**TABLE 1:** Survey domains

**Demographics** Geographic – ZIP code Individual – age, gender, race, ethnicity, educational attainment, gender, sexual orientation, employment status, health insurance status
**Health Status** Primary cancer type Date of cancer diagnosis Overall health Comorbidities
**COVID-19 Pandemic and Social Distancing** New financial stress, food insecurity Canceled or delayed health care Adherence to social distancing Willingness to receive COVID-19 vaccine, type of vaccine received
**Health Behavior** Physical activity/inactivity Fruit/vegetable intake Alcohol intake Tobacco/marijuana intake Adherence to cancer treatment or adherence to cancer surveillance

The questionnaire queried various health behaviors including physical activity (number of days in an average week they engaged in moderate and vigorous activity), fruit and vegetable consumption (number of times per day, week, or month that fruits and/or vegetables were consumed), alcohol consumption (number of drinks per day, week, or month that occurred in the last 30 days), and marijuana/tobacco use (in the last 30 days, selecting from a list with options such as cigarettes, smokeless tobacco, or dip, etc.). Those who reported consuming one or more alcoholic drink in the last 30 days were asked how many days, in the last 30 days, on which they consumed five or more drinks on the same occasion. Participants were then asked whether their participation in the respective activities increased, decreased, or stayed the same throughout the COVID-19 pandemic. Participants were asked to report their perception of their health status and identify the type(s) of cancer they were currently being or had been recently treated for.

To assess the impact of COVID-19 on access to and use of medical services we asked participants, since the start of pandemic-based restrictions in March of 2020, whether or not they had needed a medical/dental appointment, their medical/dental practice had closed, they had canceled a medical/dental appointment to avoid being around others, and they had been able to receive prescription and over-the-counter medicines. For cancer-specific care, participants were asked whether they had canceled or rescheduled cancer-related appointments and if so, the type of appointment that had been canceled or rescheduled.

Participants were asked to report their annual combined household income and highest level of education. Participants were then asked whether they had experienced job loss, work-hour reduction and/or income reduction (yes/no/unsure) since the beginning of the COVID-19 lockdown on March 25, 2020. In addition, the survey queried the participants’ ability to pay bills and purchase food and other items. The survey also assessed participants’ self-reported adherence to social distancing measures and receipt of the COVID19 vaccine.

### Statistical Analysis

We used descriptive statistics (means for continuous variables and proportions for categorical variables) to characterize the participant population. We compared the characteristics of rural and urban participants using bivariate analysis (*t* test for continuous variables, *χ*^2^ or Fisher exact tests as appropriate for categorical variables). Analyses were conducted using SAS version 9.4 (SAS Institute Inc.) software.

Data were generated by the authors and are available upon request.

## Results

### Demographics

In total, 515 rural patients with cancer and cancer survivors (43.5% of those contacted) and 146 urban patients with cancer and survivors (40% of those contacted) enrolled in the study (Table 2). About half of all rural respondents were from large rural areas (48.2%) and half from small or isolated small rural areas (51.8%). Rural respondents had a higher average age (69 years) compared with urban respondents (67 years; *P* = 0.03). Urban-rural differences were noted in insurance status: More rural respondents (94.4%) had health insurance than their urban counterparts (88.4%) (*P* = 0.01). More rural respondents had Medicare and Military/Veteran's Administration insurance (36.7% and 8.7%, respectively) than urban respondents (26.7% and 3.4%; *P* = 0.03). Rural and urban respondents were similar across remaining demographic characteristics.

**TABLE 2:** Characteristics of urban and rural patients with cancer and cancer survivors

	Rural	Urban	Total	
	*N* = 515	*N* = 146	*N* = 661	*P*
**Average age**	69.2 (11.0)	66.9 (11.1)	68.7 (11.0)	0.03
**Gender**				NS
Male	44.2%	35.9%	42.4%	
Female	54.9%	64.1%	56.9%	
Neither male nor female	0.9%	0.0%	0.9%	
**Sexual orientation**				NS
Heterosexual or straight	93.4%	94.0%	93.5%	
Gay or lesbian	2.7%	0.8%	2.3%	
Neither	0.8%	0.8%	0.8%	
Prefer not to answer	3.1%	4.5%	3.4%	
**Race**				
American Indian or Alaska Native	1.6%	2.8%	1.9%	NS
Asian	1.0%	0.7%	0.9%	NS^a^
Black or African American	0.6%	0%	0.5%	NS^a^
Native Hawaiian or Other Pacific Islander	0%	0.7%	0.2%	NS^a^
White	90.7%	88.7%	90.2%	NS
Other	2.7%	1.4%	2.4%	NS^a^
**Ethnicity**				NS^a^
Hispanic/Latino	2.3%	4.6%	2.8%	
Not Hispanic/Latino	97.7%	95.4%	97.2%	
**Education**				NS
Less than high school	1.8%	3.0%	2.1%	
GED/High school graduate	11.0%	9.7%	10.8%	
Some college, but no bachelor's degree	32.1%	38.8%	33.6%	
Bachelor's degree	31.5%	27.6%	30.7%	
Master's degree, professional school degree or doctorate	23.5%	20.9%	23.0%	
**Annual household income**				NS
$0–$19,999	10.4%	4.7%	9.1%	
$20,000–$49,999	19.4%	27.6%	21.2%	
$50,000–$74,999	19.4%	20.5%	19.7%	
$75,000–$99,999	21.6%	21.3%	21.6%	
$100,000–$199,999	25.2%	18.9%	23.8%	
$200,000 or more	4.0%	7.1%	4.7%	
**Job loss due to COVID**	5.7%	6.8%	5.9%	NS
**Work hours or income reduced because of COVID**	11.9%	13.1%	12.2%	NS
**Current household financial situation**				NS
Pay bills/special	65.2%	69.2%	66.0%	
Pay bills only	21.6%	18.5%	20.9%	
Cutting back to pay bills	9.5%	9.2%	9.5%	
Can't pay bills	3.7%	3.1%	3.6%	
**Experiencing food insecurity**	9.7%	8.4%	9.4%	NS
**Has health insurance**	94.4%	88.45	93.0%	0.01
**Type of health insurance**				
Medicaid	8.5%	6.2%	8.0%	NS
Private	23.9%	29.5%	25.1%	NS
Medicare	36.7%	26.7%	34.5%	0.03
Medicare plus a supplemental policy	42.9%	41.1%	42.5%	NS
Military/VA	8.7%	3.4%	7.6%	0.03
Other	7.4%	3.4%	6.5%	NS

NOTE: Missing values: age 49, gender 62, sexual orientation 45, race 17, ethnicity 49, education 38, income 81, occupational status 39, paid employment 38, job loss 38, work hours/income reduction 53, current household financial situation 49, food insecurity 44, health insurance 38, health insurance type 43.

^a^A Fisher exact test was used to test for significance.

### Health Status

In Table 3, among rural respondents, 43.2% reported having excellent or very good health status compared with 30.5% urban respondents, though this difference was not significant. Breast (27.6%) and prostate (15.2%) were the most common cancers rural respondents reported (Table 2). Breast cancer (31.5%), blood cancer (20.6%), and prostate cancer (9.6%) were most common among urban respondents. More rural than urban respondents reported skin cancer (12.2% vs. 3.8%; *P* = 0.01) while more urban than rural respondents reported blood cancers (20.6% vs. 12.0%, respectively; *P* = 0.01). More urban than rural respondents were reported using oral medication for cancer treatment and/or prevention (45.3% vs. 31.0%; *P* = 0.01). Most self-reported comorbidities reported in Table 3 were equally prevalent between rural and urban respondents.

**TABLE 3:** Health characteristics of participants

	Rural (%)	Urban (%)	Total (%)	Statistical
	*N* = 515	*N* = 146	*N* = 661	significance
**Perception of health status**				
Excellent/Very good	43.2%	30.5%	40.5%	NS
Good	31.5%	41.2%	33.6%	
Fair	20.3%	23.7%	21.0%	
Poor	5.1%	4.6%	5.0%	
**Cancer type**				
Breast	27.6%	31.5%	28.4%	NS
Prostate	15.2%	9.6%	13.9%	NS
Blood	12.0%	20.6%	13.9%	<0.01
Colorectal	6.0%	6.2%	6.2%	NS
Skin	12.2%	3.8%	10.4%	<0.01
Lung	5.6%	5.5%	5.6%	NS
Other	18.3%	20.5%	18.8%	NS
**Currently on oral medication for cancer treatment/cancer prevention**	31.0%	45.3%	34.2%	<0.01^a^
**Comorbidities**				
Heart disease	15.5%	11.6%	14.7%	NS
Lung disease	11.8%	9.6%	11.4%	NS
Ulcer or stomach disease	4.7%	2.7%	4.2%	NS
Liver disease	4.1%	4.1%	4.1%	NS
Osteoarthritis or degenerative arthritis	22.7%	13.7%	20.7%	0.02
Rheumatoid arthritis	4.5%	2.7%	4.1%	NS
Depression	15.7%	19.2%	16.5%	NS
High blood pressure	44.5%	38.4%	43.1%	NS
Diabetes	13.2%	13.0%	13.2%	NS
Kidney disease	7.2%	5.5%	6.8%	NS
Anemia or other blood disease	9.3%	10.3%	9.5%	NS
Back pain	30.1%	25.3%	29.1%	NS
HIV	0.4%	0%	0.3%	NS^a^
Other	31.7%	28.8%	31.0%	NS
None	12.8%	11.0%	12.4%	NS

NOTE: Missing values: participant rating of health status 41, currently on oral medication 82, other cancer 85.

^a^A Fisher exact test was used to test for significance.

### Health Behaviors

Rural respondents on average consumed more alcohol per week than their urban counterparts (4.4 vs. 2.7 drinks per week, respectively (*P* < 0.01; see Table 4). There were no significant differences in mean number of days of exercise per week (3.6 days/week overall) or of fruits and vegetables eaten (2.1 servings per day) between urban and rural respondents. There was no difference among rural and urban respondents in the proportion who reported an increase in alcohol consumption (12.7% rural vs. 10.1% urban) or an increase in tobacco consumption (13.2% rural vs. 14.3% urban; Table 4).

**TABLE 4:** Average occurrence of and self-reported changes in cancer-related behaviors by rural and urban patients with cancer/survivors since the start of the COVID-19 pandemic

	Rural	Urban	Total	Statistical
	*N* = 515	*N* = 146	*N* = 661	significance
Mean number of alcoholic drinks/week	4.4	2.7	4.1	<0.01
Mean days exercise/week (answer > 7 set to missing)	3.6	3.5	3.6	NS
Mean servings of fruit and vegetables eaten/day	2.0	2.1	2.1	NS
**Change in health behaviors**				
**Alcohol consumption**				
Increased alcohol consumption^a^	12.7%	10.1%	12.2%	NS
Decreased alcohol consumption^a^	15.1%	24.6%	17.0%	
No change in alcohol consumption^a^	72.2%	65.2%	70.8%	
**Tobacco consumption**				
Increased tobacco consumption^a^	13.2%	14.3%	13.3%	NS
Decreased tobacco consumption^a^	14.5%	14.3%	14.5%	
No change in tobacco consumption^a^	72.4%	71.4%	72.3%	
**Physical activity**				
Decreased physical activity	38.2%	39.5%	38.5%	NS
Increased physical activity	8.6%	12.4%	9.4%	
No change in physical activity	53.2%	48.1%	52.1%	
**Fruit/vegetable consumption**				
Decreased fruit and vegetable consumption	7.3%	5.3%	6.9%	NS
Increased fruit and vegetable consumption	8.5%	15.3%	10.0%	
No change in fruit and vegetable consumption	84.2%	79.4%	83.2%	

NOTE: Missing values: weekly alcoholic drinks 85, exercise days/week 97, exercise days/week imputed 45, daily fruit and vegetable servings 54, alcohol perception 5, tobacco use perception 40, Exercise perception 38, fruit and vegetable perception 40.

^a^Only those who reported using alcohol (*n* = 353) were asked about their change in alcohol use: only responses from those who reported using tobacco were included in the tobacco consumption question (*n* = 123).

### Health Care Utilization

Nearly all rural (94.7%) and urban (95.5%) participants noted that they had needed a medical or dental appointment of some kind since March 2020. A total of 36.0% of rural and 42% of urban residents experienced a medical or dental practice closure during the COVID19 pandemic, though this difference was not statistically significant. There was no difference in proportions of rural versus urban respondents that reported canceled or delayed cancer care (19.6% rural, 23.4% urban; Table 5).

**TABLE 5:** Percentages of urban and rural patients with cancer/survivors that reported canceled or delayed health care since the start of the COVID-19 pandemic

	Rural (%)	Urban (%)	Total (%)	Statistical
	*N* = 515	*N* = 146	*N* = 661	significance
Appointment needed since March 25, 2020	94.7%	95.5%	94.9%	NS
Experienced medical/dental practice closure	36.0%	42.1%	37.3%	NS
Canceled medical/dental appointment	29.0%	23.0%	27.8%	NS
Unable to obtain prescription medication	5.8%	4.6%	5.6%	NS
Unable to obtain over-the-counter medication	5.1%	8.6%	5.8%	NS
Canceled/delayed cancer care	19.6%	23.4%	20.5%	NS
Type of cancer care delayed				
Routine appointment	15.7%	17.1%	16.0%	NS
Screening test	8.0%	4.1%	7.1%	NS
Blood test	5.4%	6.2%	5.6%	NS
Surgery	3.9%	2.7%	3.6%	NS
Chemotherapy	1.8%	3.4%	2.1%	NS^a^
Radiotherapy	1.6%	1.4%	1.5%	NS^a^
Therapy (physical or occupational)	1.6%	1.4%	1.5%	NS^a^
Other	8.7%	7.5%	8.5%	NS

NOTE: Missing values: health appointment needed 36, experienced medical/dental practice closure 68, canceled medical/dental appointment 70, unable to obtain prescription medication 37, unable to obtain OTC medication 36, cancelled/delayed cancer care 85.

^a^A Fisher exact test was used to test for significance.

Both rural and urban respondents reported high levels of adherence to social distancing recommendations (Table 6). Almost all (97.4%) said they wore a face mask indoors when they were not at home, kept 6 feet between themselves and others (90.1%), and stayed home except for necessities (85.2%). These actions were consistent with respondents’ perceptions of the importance of social distancing: three-quarters (74.6%) said it was very important and 16.3% felt it was somewhat important. Among those surveyed after the COVID-19 vaccine was available, more rural respondents (91.2%) than urban respondents (82.1%) indicated they had received it (*P* = 0.04).

**TABLE 6:** Vaccine receipt and social distancing tendencies of rural versus urban patients with cancer/survivors in Washington and Idaho from January to June 2021

	Rural (%)	Urban	Total	Statistical
	*N* = 515	*N* = 146	*N* = 661	significance
Received COVID-19 vaccine^a^	91.2%	82.1%	89.6%	0.04
Staying home except for necessities	85.9%	82.8%	85.2%	NS
Having friends/relative visit the home	42.1%	41.8%	42.0%	NS
Keeping 6 feet between self and others	90.1%	90.1%	90.1%	NS
Wearing face covering outdoors	47.9%	45.9%	47.5%	NS
Wearing face mask indoors when not at home	96.9%	99.3%	97.4%	NS
Attended indoor gathering	39.1%	41.0%	39.5%	NS
Attended 20+ person gathering	4.1%	3.0%	3.9%	NS

NOTE: Missing values: staying home 35, relative/friends visit the home 38, social distancing 6 feet 39, face covering outdoors 37, face covering indoors when not at home 37, attended indoor gathering 36, attended large gathering 39.

*^a^* The COVID-19 vaccine was not available for the entire time this survey was administered, which resulted in 324 of the 661 respondents being asked this question.

## Discussion

In this cross-sectional survey assessing the impact of COVID19 on health behaviors, we found few differences in changes in reported health behaviors among rural and urban patients with cancer and cancer survivors. The mean number of alcoholic drinks was higher among rural respondents. Rural residence is associated with higher rates of alcohol consumption in patients with head and neck cancer (19), yet rural residents are less likely to receive evidence-based alcohol screening and intervention in primary care (20). A total of 12.2% of respondents overall reported an increase in alcohol consumption since the start of the pandemic. While this is lower than the proportion of the general population that reported an increase in alcohol consumption during the same time frame (6), the potentially negative health consequences of increased alcohol consumption in patients with cancer and cancer survivors is of great concern; Alcohol consumption among patients with cancer is associated with higher cancer-related mortality (21–23).

Overall rates of physical activity were low, and a sizeable proportion in both rural and urban respondents reported decreased physical activity since the start of the pandemic. Nationally, during the COVID-19 pandemic, the general population also reported a decrease in physical activity (24) and overall weight gain (25). In patients with cancer, physical activity reduces fatigue, decreases reported side effects of some cancer treatments, and improves functional outcomes (26–29). Access to tailored activity programs is an important facilitator of regular physical activity in patients with cancer and cancer survivors (30, 31). The COVID-19 pandemic, which led to closures and delays of many community-based programs, likely contributed to decreased access to physical activity programs and opportunities for this population.

A total of 12.2% of respondents overall reported an increase in alcohol consumption since the start of the pandemic. While this is lower than the proportion of the general population that reported an increase in alcohol consumption during the same time frame (6), the potentially negative health consequences of increased alcohol consumption in patients with cancer and cancer survivors is of great concern; alcohol consumption among patients with cancer is associated with higher cancer-related mortality (21–23).

We found that 20% of respondents reported canceling or delaying cancer care due to COVID-19. Delays in surgeries, chemotherapy, and radiotherapy could have profound impact on cancer outcomes. For rural populations, missed radiation treatments appear to contribute to excess cancer-mortality observed in rural communities (32). Health care systems, including cancer centers, rapidly switched to offering care remotely via telemedicine at the start of the COVID-19 pandemic (33–35). However, patients with cancer and oncologists report a preference for in-person visits (36), and critical cancer therapies, such as surgeries and chemotherapy, cannot be delivered through telemedicine. Combining telemedicine and in-person care when needed or preferred and addressing COVID-19 transmission concerns through social distancing and screening measures could help ensure that patients with cancer continue to receive optimal care while reducing risk of COVID-19 infection.

We found that rates of adherence to social distancing and uptake of COVID-19 vaccination were high in both rural and urban respondents. Nationally, as of August 11, 2021, the rate of adult COVID-19 vaccination in rural counties was only 46%, compared with 60% in urban counties (37), but rates of COVID-19 vaccination are higher overall, even in rural communities, among older adults (38) and among individuals with serious medical conditions such as cancer (39). A national effort by cancer centers to support early vaccination of patients with cancer and survivors (40) may have contributed to early and rapid uptake of COVID-19 vaccination in our study population.

This study has several potential limitations. All information was collected directly from respondents, and self-reported measures may be subject to bias. Participants may have been more likely to report positive behavior changes than negative behavior changes, leading to an underestimate of the negative health consequences of the COVID-19 pandemic. Respondents may differ in important characteristics compared with nonrespondents, but no information about nonrespondents was available. Finally, this study used bivariate analysis to compare urban and rural patients with cancer and cancer survivors. Adjusted analysis may help explore non-geographic factors which may be associated with changes in self-reported health behaviors among this population but was beyond the scope of this study. Despite this, our sample was obtained from two states, and our partnership with six cancer centers and oversampling of rural participants allowed us to explore important rural-urban differences in health behaviors and health care access during the COVID19 pandemic.

## Conclusions

Though few differences between urban and rural patients with cancer and cancer survivors were detected when exploring the impact of COVID19 on health behaviors and health care utilization, we did find that rural patients with cancer and cancer survivors reported higher average alcohol consumption. Our findings also contribute to an understanding of the impact of COVID19 on patients with cancer and cancer survivors, generally, with reported decreased physical activity, increased alcohol consumption, and increased tobacco consumption. Respondents also reported delaying or canceling cancer care because of the pandemic. These changes in health behaviors and health care utilization, if sustained, could have a significant negative impact on cancer health outcomes.
